# Supplementary material for: Understanding molecular mechanisms and predicting phenotypic effects of pathogenic tubulin mutations
Source: PLoS Comput Biol. 2022 Oct 7;18(10):e1010611. doi: 10.1371/journal.pcbi.1010611 (PMC9581425; doi:10.1371/journal.pcbi.1010611)
Supplement: S1 Text — File containing additional figures complementary to the analysis shown in the main text of this manuscript. (DOCX) [file pcbi.1010611.s001.docx]

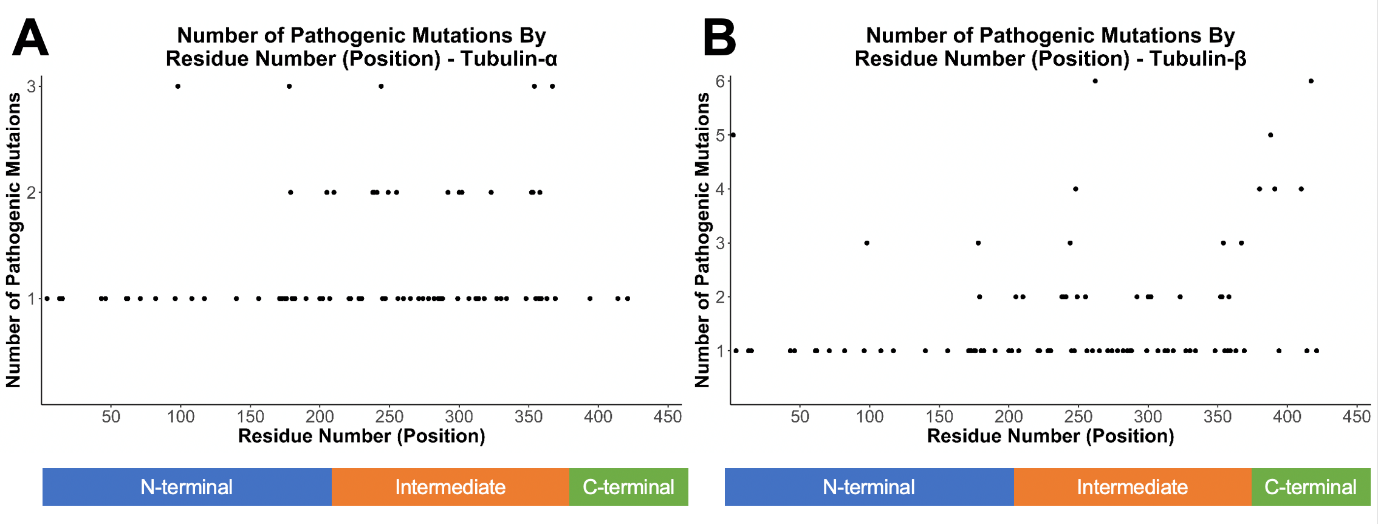


**Figure A: Occurrence of pathogenic mutations in tubulin.**

Number of pathogenic mutations according to the residue position for tubulin-α **(A)** and β **(B)**.


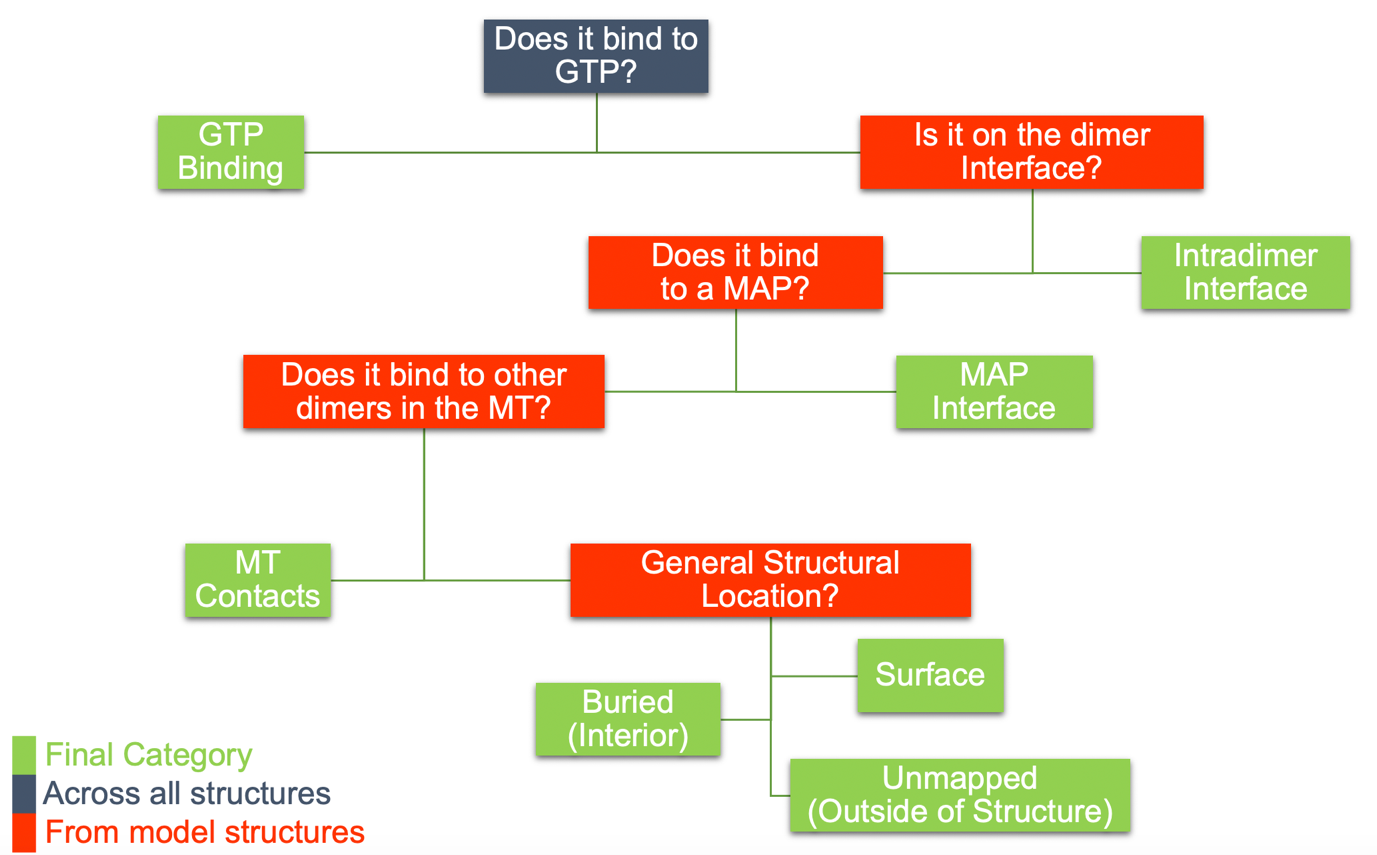


**Figure B: Hierarchy diagram for classification of structural locations.**

Schematic illustrating the hierarchy used for classification of structural locations, indicating where all structures with 70% protein sequence identity were considered, or where specific model structures were used (according to S2 Table).


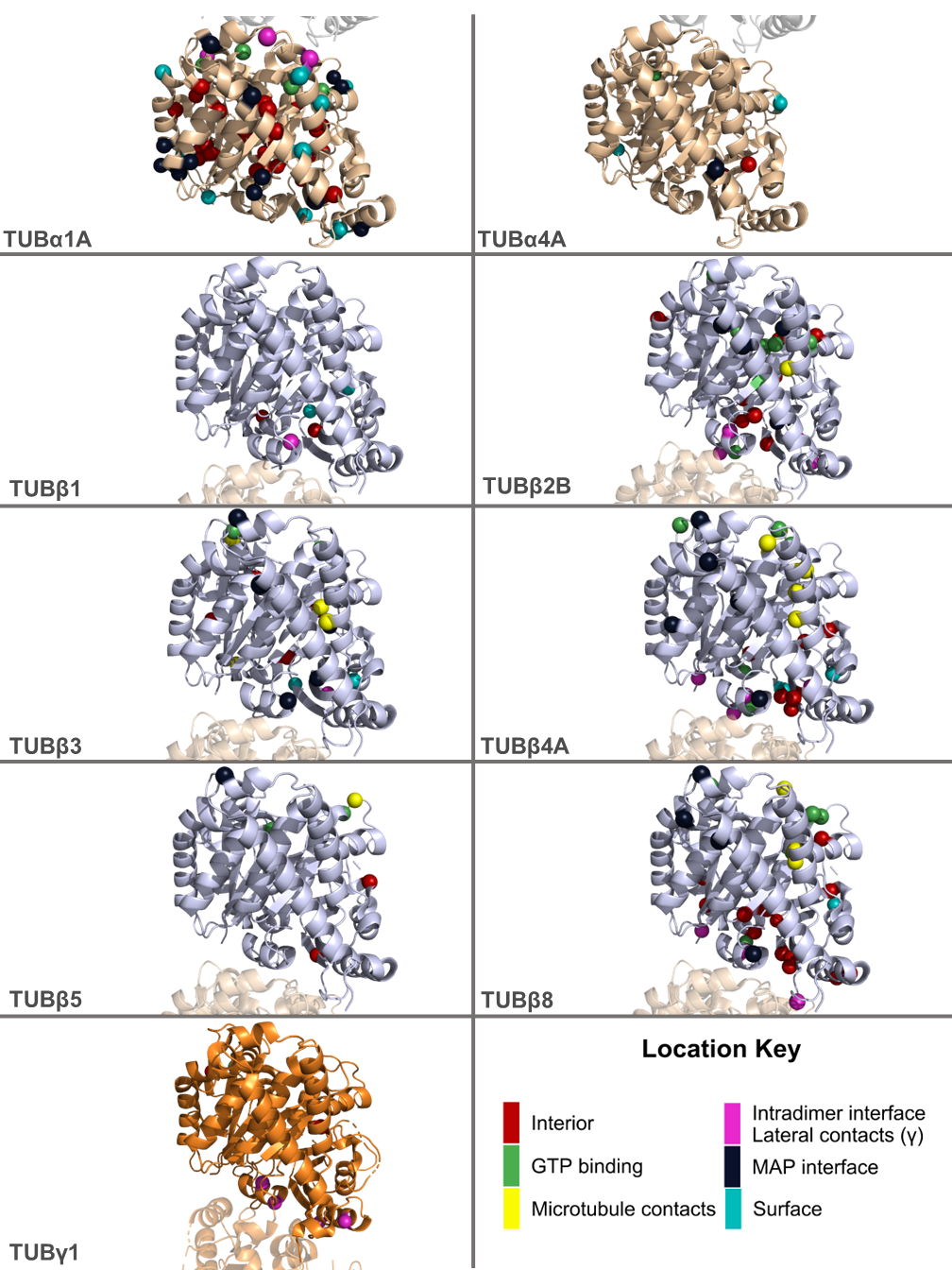


**Figure C: Visualisation of pathogenic tubulin mutations on three-dimensional structures of tubulin heterodimers (90° rotation).**

Same as Figure 2, but structures are rotated 90° around the y-axis.


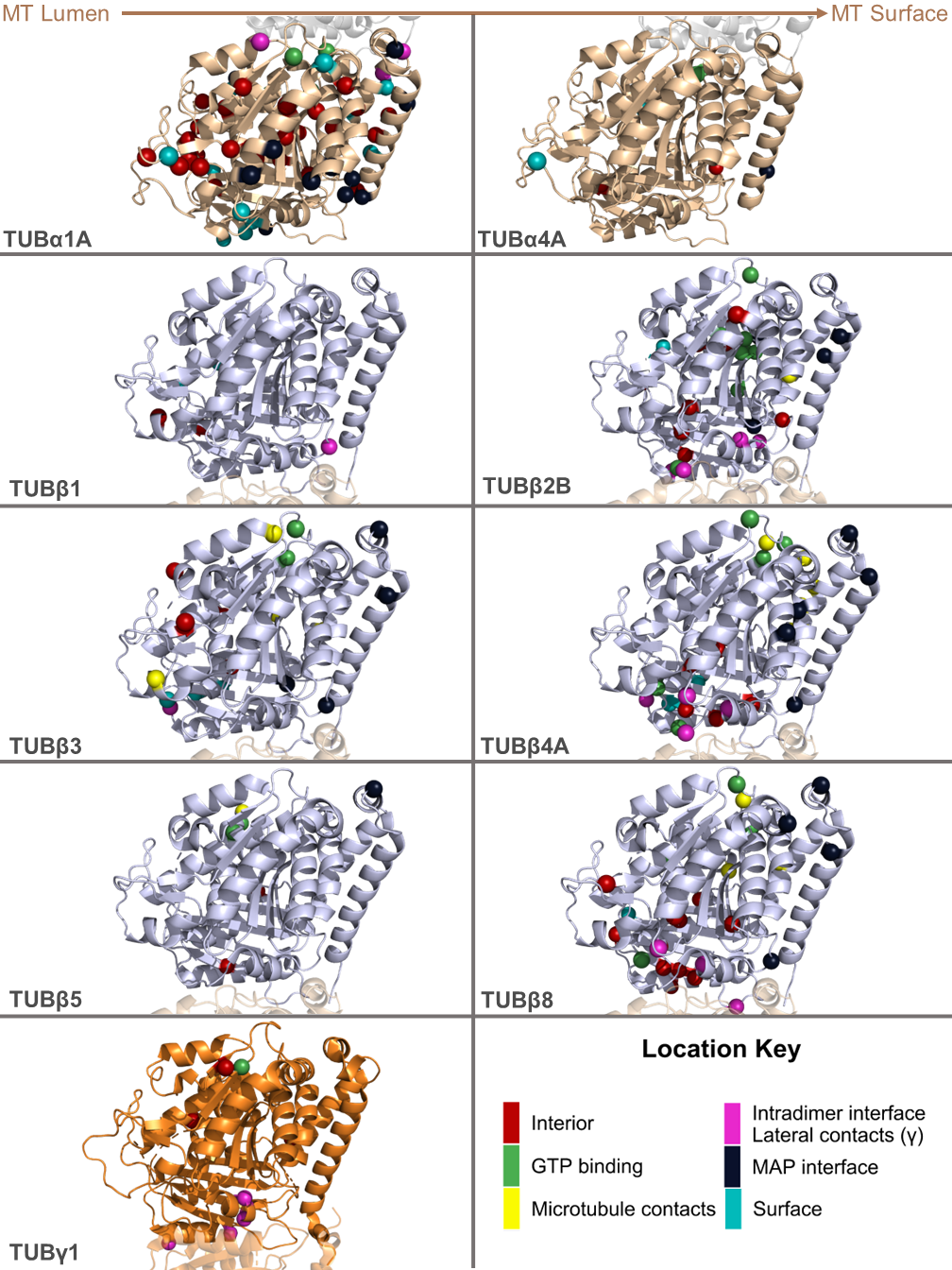


**Figure D: Visualisation of pathogenic tubulin mutations on three-dimensional structures of tubulin heterodimers (180° rotation).**

Same as Figure 2, but structures are rotated 180° around the y-axis.

**
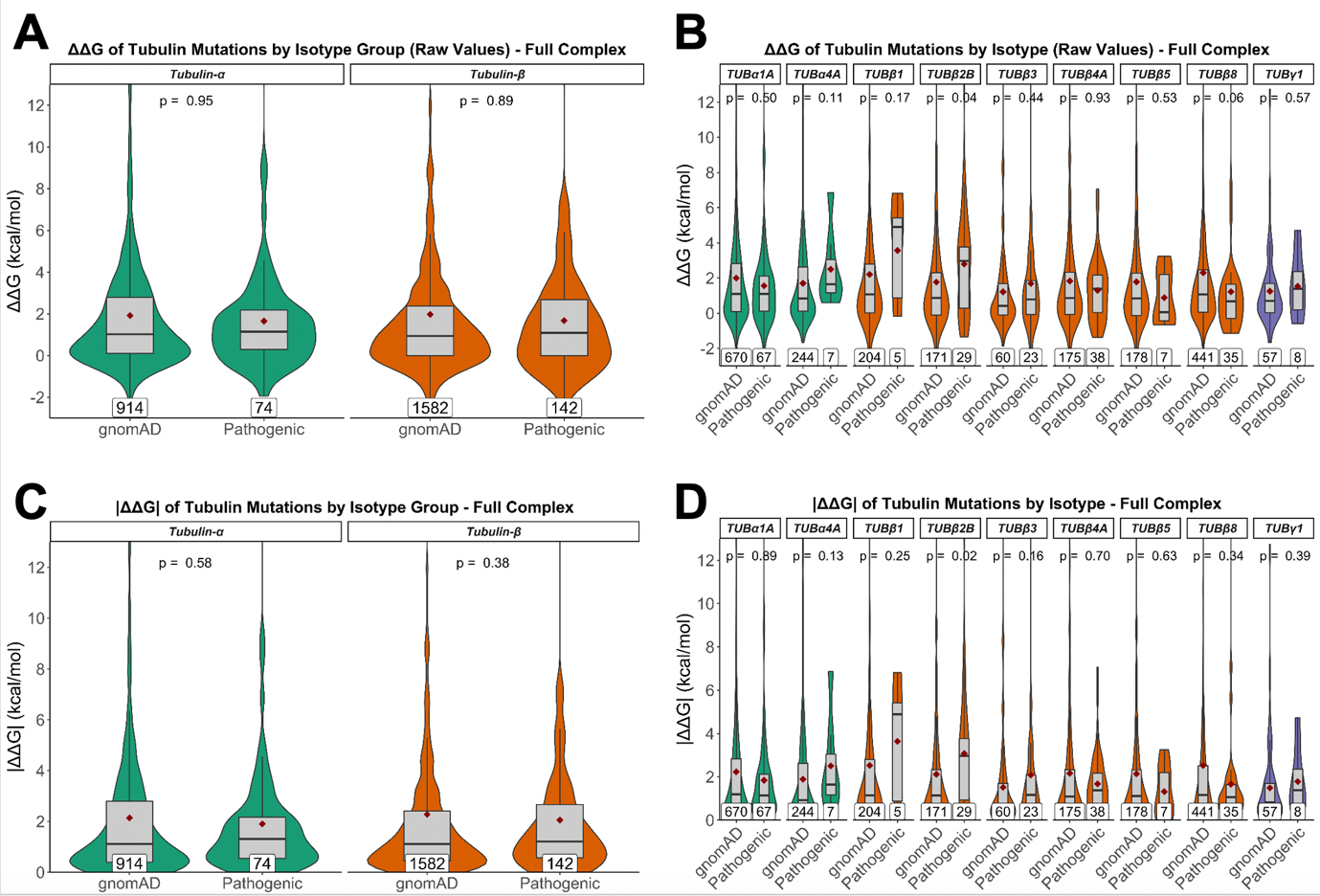
**

**Figure E: Comparison of predicted changes in protein stability between pathogenic and putatively benign tubulin variants, when considering ΔΔG values calculated using full protein complex structures and absolute values**.

ΔΔG values calculating the change in free energy for folding were calculated with FoldX considering the structure of the full protien complex structures, including all intermolecular interactions. Scores are shown for tubulin-α and β families globally **(A)**, and in isotypes with at least 5 identified pathogenic mutations **(B)**. Absolute values for these scores are also shown for each family **(C)** and isotype **(D)**. Maroon diamonds indicate the mean ΔΔG score, and mutation totals for each group are also shown at the bottom. The p-values displayed were obtained via unpaired Wilcoxon tests.

**
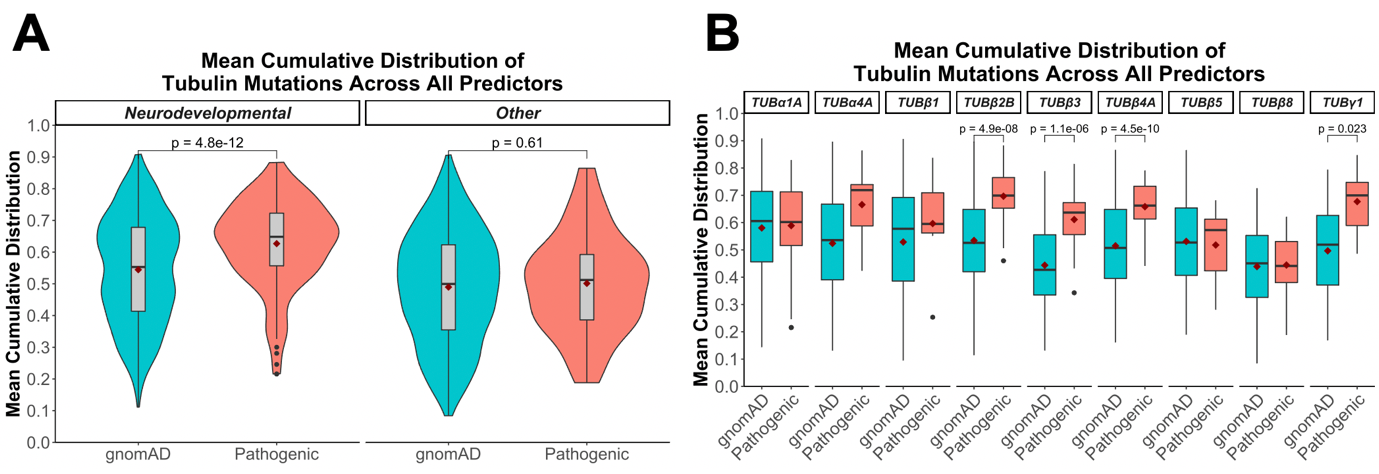
**

**Figure F: Calculating the MCD scores across all predictors for each mutation reveals similar patterns**.

MCDs were calculated across all VEPs in a combined dataset which included all gnomAD and pathogenic mutations across all tubulins. The gnomAD and pathogenic MCD scores were separated and grouped by pathogenicity type **(A)**, or isotype **(B)**. The p-values stated were obtained using unpaired Wilcoxon tests, with ones not shown being above 0.05. Maroon diamonds indicate the average MCD.

**
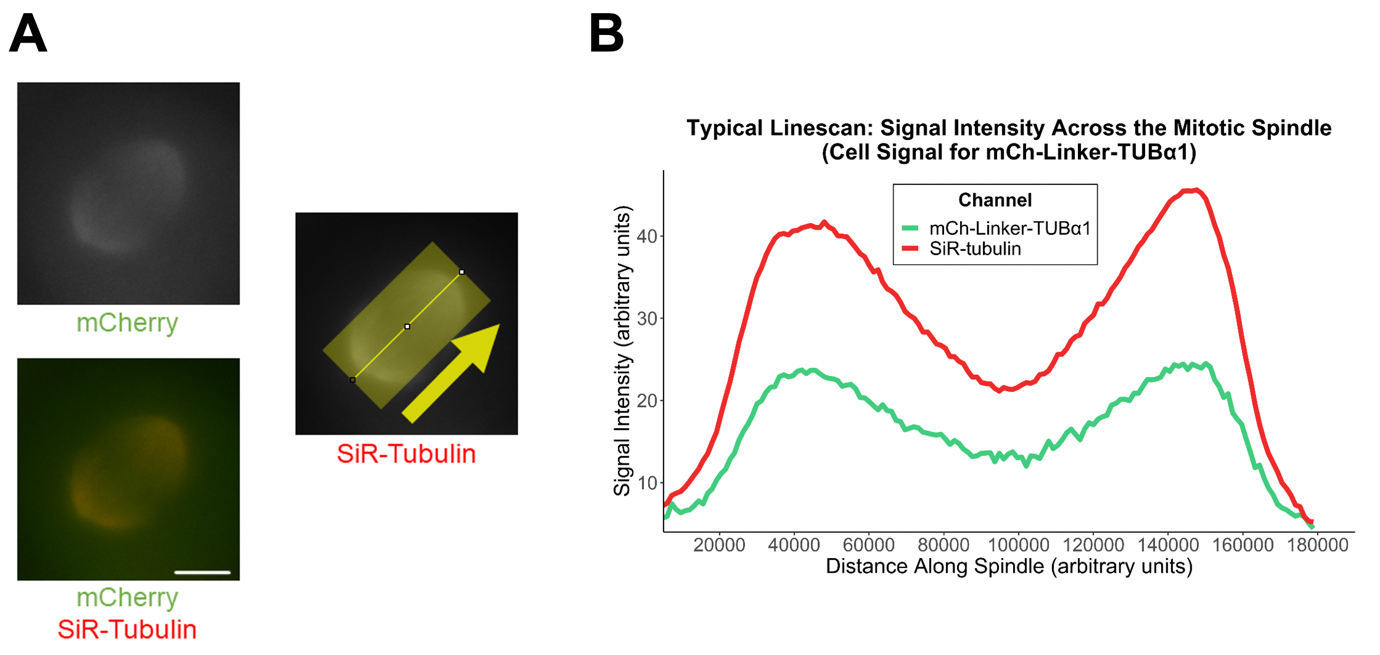
**

**Figure G: Typical linescan workflow.**

**(A)** Representative live-cell images of mitotic HeLa cell after being transfected with fluorescently tagged wild type mCherry-Linker-TUBα1 (green) and incubated with SiR-tubulin (red). Using the SiR-tubulin channel, we take a wide linescan (yellow, arrow indicating direction of linescan along the x-axis) of the mitotic spindle and do the same for the fluorescent channel in each cell to determine signal intensity **(B)**. This is measured by the average background-subtracted grey value of each point across the wide line. The spearman correlation coefficient of these two traces along the linescan are then calculated on a cell-by-cell basis to account for differences of intensity.

**
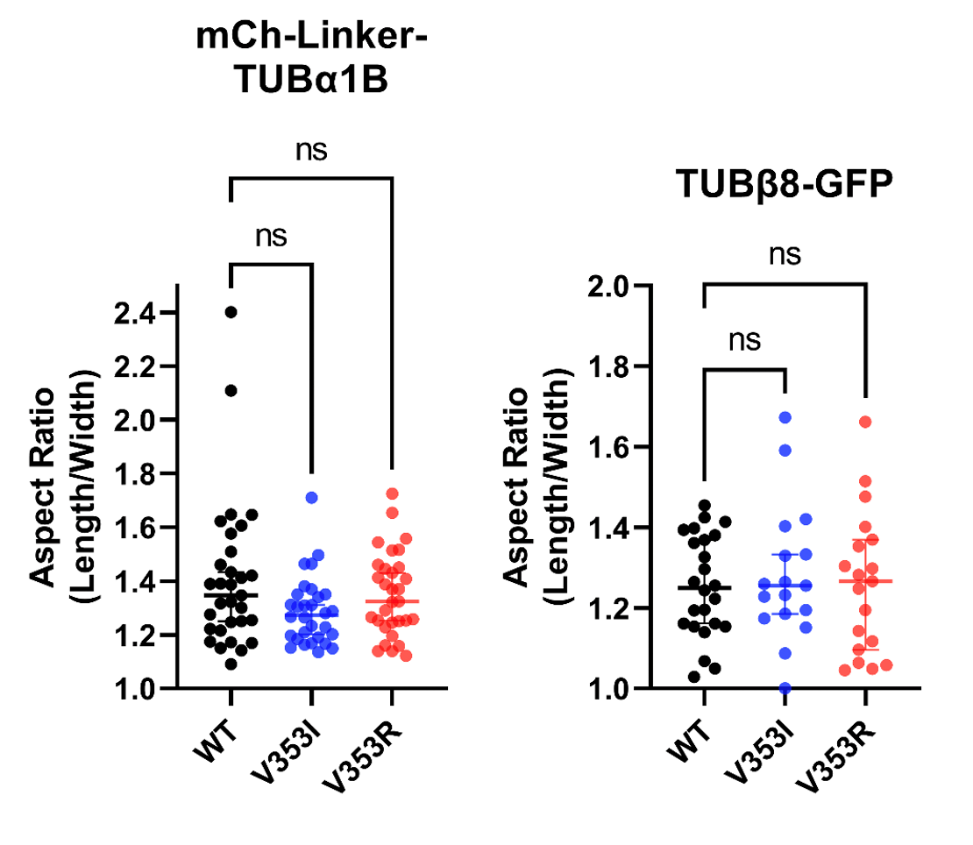
**

**Figure H: Mitotic spindle width and aspect ratio of V353 mutants in TUBα1 and TUBβ8.**

HeLa cells were transfected with fluorescently tagged wild type and mutant constructs and incubated with SiR-tubulin (red). With each construct, the spindle aspect ratio (length/width) was calculated for each cell. Kruskal-Wallis tests (with post-hoc Dunn) were performed, but no significant differences were found.


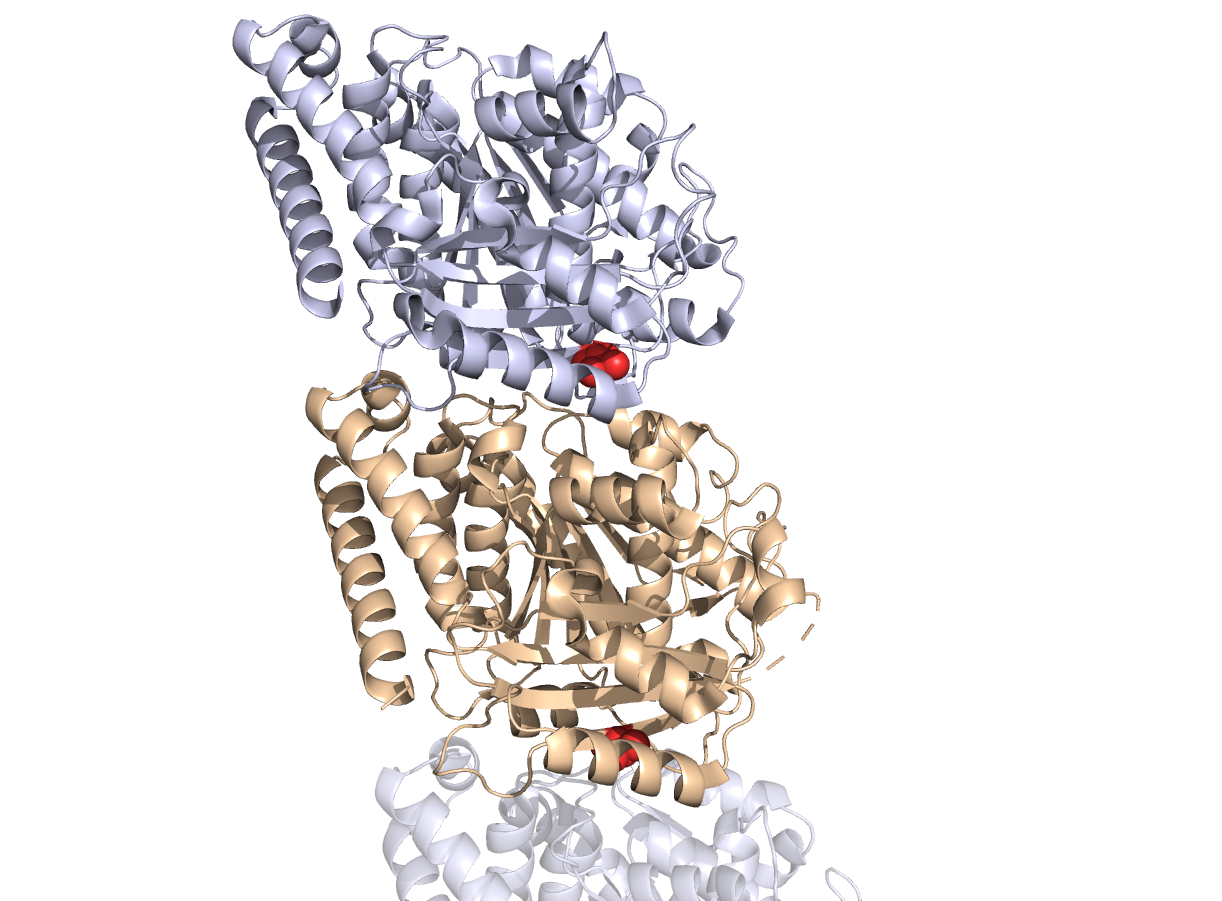


**Figure I: Visualisation of V353 residues in tubulin-α and β.**

Tubulin-α is in wheat, while tubulin-β in bluewhite. Red spheres indicate atoms constituting the V353 residues in both proteins. Part of the next heterodimer along the microtubule protofilament is also shown in reduced transparency to show the adjacency of the V353 residue in tubulin-α to the longitudinal interdimer interface. PDB ID: 5jco
