# Supplementary material for: Understanding molecular mechanisms and predicting phenotypic effects of pathogenic tubulin mutations
Source: PLoS Comput Biol. 2022 Oct 7;18(10):e1010611. doi: 10.1371/journal.pcbi.1010611 (PMC9581425; doi:10.1371/journal.pcbi.1010611)
Supplement: Attachment — Submitted filename: review_attard_welburn_marsh.pdf [file pcbi.1010611.s010.pdf]

Significance & General Interest

Tubulinopathies, disorders associated with a disfunction of the microtubule cytoskeleton in cells, give rise to a large number of phenotypes, most of which have no severe hallmarks that can be easily characterized using common methods. This is not surprising given the immense importance of microtubules for cellular dynamics and organization — a mutation that disrupts the core structure of tubulin or prevents tubulin heterodimer formation would have immediate lethal consequences at a very early developmental stage of an organism. Therefore, many pathogenic tubulin mutations affect microtubules at the levels of either their biophysical properties (e.g., polymerization properties) or their "controllability" (i.e. the ability of the cell to efficiently organize microtubules using a plethora of microtubule-associated proteins (MAPs)). The study by Attard et al. is a timely and important survey and classification of tubulin mutations, which also identifies potential pitfalls in state-of-the-art prediction methods and is supported by experiments for one characteristic mutation.

Quality of Work

Overall, the quality of work is reasonably high. The representation of tubulin 3D structures and in Fig. 2, in my view, does not allow to fully correlate the location of the mutations and the specific interfaces.

Conclusions Justified?

Yes (see below).

Recommendations

Minor revision.

Although the incorporation of the V353 mutants into mitotic spindles is a valid approach to assess the effects of this mutation, the experimental setup is likely not sensitive enough to detect those. Furthermore, analysis of static live-cell images does not fully reflect potential dynamic deficits these mutant microtubules might have. Having dynamic data for V353 or any other gain-of-function mutant would be nice in my opinion (e.g., a simple polymerization assay to quantify assembly/disassembly rates and/or catastrophe frequencies) and better support the authors' conclusions that gain-of-function mutants tune the dynamics of microtubules and/or interactions with MAPs rather than disrupt its basic function (i.e., the ability to form intact microtubule lattices from correctly folded and formed tubulin dimers).
