# Supplementary material for: Understanding molecular mechanisms and predicting phenotypic effects of pathogenic tubulin mutations
Source: PLoS Comput Biol. 2022 Oct 7;18(10):e1010611. doi: 10.1371/journal.pcbi.1010611 (PMC9581425; doi:10.1371/journal.pcbi.1010611)
Supplement: Attachment — Submitted filename: response.docx [file pcbi.1010611.s011.docx]

**Response to Reviewers**

**Reviewer #1**

***Alpha Tubulin and beta tubulin, which make up microtubules, have been linked to a variety of disorders that are often dominant, sporadic, and congenital. Although the first identified tubulin mutations are involved with neurodevelopment, mutations are also linked to other conditions, such as bleeding disorders and infertility. The authors observe that the co-localization of few tubulin isotype mutations with the mitotic spindle in HeLa cells suggests that they may exhibit dominant-negative effects through affecting microtubule characteristics. The authors suggest that they constitute a blind area for existing computational methods, since they are less accurately predicted than mutations in the majority of human diseases. Their computational supporting data supports their conclusions. There have been very few computational studies conducted on tubulin isotypes and their function. This work provide some novel insights on tubulin mutations and their role in pathogenicity which could be further tested in future through experiments.***

We are thankful to the reviewer for their positive comments, and their recognition of the importance of this work.

**Reviewer #2**

***Significance & General Interest**

***Tubulinopathies, disorders associated with a disfunction of the microtubule cytoskeleton in cells, give rise to a large number of phenotypes, most of which have no severe hallmarks that can be easily characterized using common methods. This is not surprising given the immense importance of microtubules for cellular dynamics and organization — a mutation that disrupts the core structure of tubulin or prevents tubulin heterodimer formation would have immediate lethal consequences at a very early developmental stage of an organism. Therefore, many pathogenic tubulin mutations affect microtubules at the levels of either their biophysical properties (e.g., polymerization properties) or their "controllability" (i.e. the ability of the cell to efficiently organize microtubules using a plethora of microtubule-associated proteins (MAPs)). The study by Attard et al. is a timely and important survey and classification of tubulin mutations, which also identifies potential pitfalls in state-of-the-art prediction methods and is supported by experiments for one characteristic mutation.***

***Quality of Work**

***Overall, the quality of work is reasonably high. The representation of tubulin 3D structures and in Fig. 2, in my view, does not allow to fully correlate the location of the mutations and the specific interfaces.***

We agree that it can be difficult to visualise the structural context of so many different mutations across several different tubulin isotypes in a single image. Therefore, we have now generated two new versions of Fig 2 where the structures have been rotated by 90 and 180 degrees around the y axis, which have been provided as Figs C-D in S1 Text. We think that, combined, these images make it much easier to comprehend the relative structural locations of the pathogenic missense mutations. We have also slightly updated Fig 2 to correct some offset mutations from the original version.

***Conclusions Justified?**

***Yes (see below).***

***Recommendations**

***Minor revision.***

***Although the incorporation of the V353 mutants into mitotic spindles is a valid approach to assess the effects of this mutation, the experimental setup is likely not sensitive enough to detect those. Furthermore, analysis of static live-cell images does not fully reflect potential dynamic deficits these mutant microtubules might have. Having dynamic data for V353 or any other gain-of-function mutant would be nice in my opinion (e.g., a simple polymerization assay to quantify assembly/disassembly rates and/or catastrophe frequencies) and better support the authors’ conclusions that gain-of-function mutants tune the dynamics of microtubules and/or interactions with MAPs rather than disrupt its basic function (i.e., the ability to form intact microtubule lattices from correctly folded and formed tubulin dimers).***

We fully acknowledge the limitations of the experiments performed in this study, and agree that further experiments providing dynamic data for V353 and other mutants would be ideal, and would provide deeper insight into molecular disease mechanisms. This is a direction that we intend to pursue in the future. However, we believe that this goes beyond the scope of the current manuscripts In addition, such follow up experiments would take several months, which is not compatible with the 30-day revision time requested by the journal, and the fact that Attard’s PhD studentship is about to end. We have now added a couple of new sentences to the Discussion addressing the limitations of our experiments and the potential for future work.
